# A Slow Axon Antidromic Blockade Hypothesis for Tremor Reduction via Deep Brain Stimulation

**DOI:** 10.1371/journal.pone.0073456

**Published:** 2013-09-16

**Authors:** Míriam R. García, Barak A. Pearlmutter, Peter E. Wellstead, Richard H. Middleton

**Affiliations:** 1 Hamilton Institute, National University of Ireland Maynooth, Co. Kildare, Ireland; 2 Department of Computer Science, National University of Ireland Maynooth, Co. Kildare, Ireland; 3 Centre for Complex Dynamic Systems & Control, The University of Newcastle, Newcastle, Australia; McGill University, Canada

## Abstract

Parkinsonian and essential tremor can often be effectively treated by deep brain stimulation. We propose a novel explanation for the mechanism by which this technique ameliorates tremor: a reduction of the delay in the relevant motor control loops *via* preferential antidromic blockade of slow axons. The antidromic blockade is preferential because the pulses more rapidly clear fast axons, and the distribution of axonal diameters, and therefore velocities, in the involved tracts, is sufficiently long-tailed to make this effect quite significant. The preferential blockade of slow axons, combined with gain adaptation, results in a reduction of the mean delay in the motor control loop, which serves to stabilize the feedback system, thus ameliorating tremor. This theory, without any tuning, accounts for several previously perplexing phenomena, and makes a variety of novel predictions.

## Introduction

About 60–70% of patients with idiopathic Parkinson's disease (PD) exhibit tremor, usually both resting and postural [1], [2]. It is believed that this pathological motor oscillation originates in the cortico-basal ganglia-thalamocortical or cerebello-thalamo-cortical motor circuits, but the precise details are unknown [3]. Nonetheless, both Parkinsonian and essential tremor have been successfully treated using a surgical technique called Deep Brain Stimulation. DBS involves stimulating certain nuclei in the ganglia-thalamo-cortical pathway with a train of high frequency (HF) (typically above 120 Hz) electrical pulses [4]. However, the fundamental question of why this technique is effective remains unresolved.

There are a number of hypotheses [5], [6], and numerous experiments have been conducted to test them, but the results have been inconclusive [6]–[9]. There are two main problems: (i) the lack of specific testable experimental predictions associated with the hypotheses, and (ii) the fundamental difficulty in explaining certain basic features of DBS. For example, why is it that only DBS at frequencies much higher than the tremor frequencies reduce tremor? And why is the therapeutic frequency range so wide? Other incompletely explained phenomena include the location of the electrode, and the observation that once DBS is activated tremor is suppressed within seconds. Despite the basic nature of these questions, they are often only considered in a peripheral manner [5], [6]. There have been a few attempts to explain the need for HF stimulation, using either large computational models with tuned parameters [10] or classical control theory [11].

Since both ablation and HF stimulation of certain parts of the deep brain structures can suppress some symptoms of PD, the first line of research was based on the *direct inhibition* hypothesis: that DBS works by reducing neuronal activity within the stimulated target [4]. In fact, the over-activity of globus pallidus internus (GPi) due to over-activity of the subthalamic nucleus (STN) provided an explanation of why local inhibition of GPi or STN should be therapeutic.

More recent observations have called the *direct inhibition* hypothesis into question. We have the apparent contradiction that lesioning of the globus pallidus externus (GPe) can produce Parkinsonism while DBS of the GPe can reverse Parkinsonian symptoms [12]. Recordings in downstream structures have demonstrated changes indicative of activation of outputs from the stimulated structures [5], [13], [14] or different effects upon corticostriatal afferents [15]. This has led to the alternative hypothesis that DBS works by introducing exogenous activity into the network, which modifies pathological spontaneous activity in a number of nuclei. Based upon this hypothesis, a number of mechanisms have been proposed, whose details depend on the relevant activated element (efferents, afferents, and/or nearby fibers) or on the observed effect in the basal ganglia (BG) network. Examples include “jamming” of abnormal patterns, firing regularization, and desynchronization of the neural network [14], [16], [17].

We hypothesize that DBS ameliorates tremor by shortening the communication delay in the cortico-basal ganglia-thalamocortical feedback loop, thus stabilizing the motor control loop [18]. This explains the problematic phenomena discussed above, while bringing a control system perspective to the DBS problem.

## Results

### Preliminaries and required assumptions

“Exactly how DBS exerts its therapeutic effects is a matter of controversy” [7]. The high therapeutic pulse frequencies of DBS, and characteristic features such as the fact that, once activated, DBS reduces tremor amplitude while increasing tremor frequency within seconds, are both difficult to account for with current theories.

One of the main reasons for the controversy surrounding the working mechanism of DBS is the difficulty of identifying neuronal elements activated by DBS that are also capable of explaining experimental results. In recent years, some groups have suggested that these results can be understood by assuming that DBS stimulates neuronal axons and not somas [5], [13]. The chronaxies of myelinated fibers vary in the range of 30–200 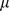 s, while cell bodies have chronaxies in the 1–10 ms band [19]. Since the usual pulse width in DBS is between 60–450 *μ*s (with more current required for the smallest widths), the longer myelinated axons connecting different structures would tend to be activated, rather than the cell bodies [5].

In the case of unmyelinated axons, the experimental estimates of chronaxies and rheobases of such fibers [19] are somewhat controversial. Mindful of this controversy, we assume that only the myelinated fibers [5] are activated but that the unmyelinated ones are not excited by the stimuli. This is also supported by the estimations of chronaxies [19], usually larger than the DBS pulse width. It should be noted that if this is the case, the beneficial effects of DBS can be attributed to the excitation of long axons connecting different parts of the brain, whilst the effects inside the stimulation structure are of limited relevance. Although more evidence in this regard would be necessary, recent experiments seem to support this hypothesis [7].

**Figure 1:**
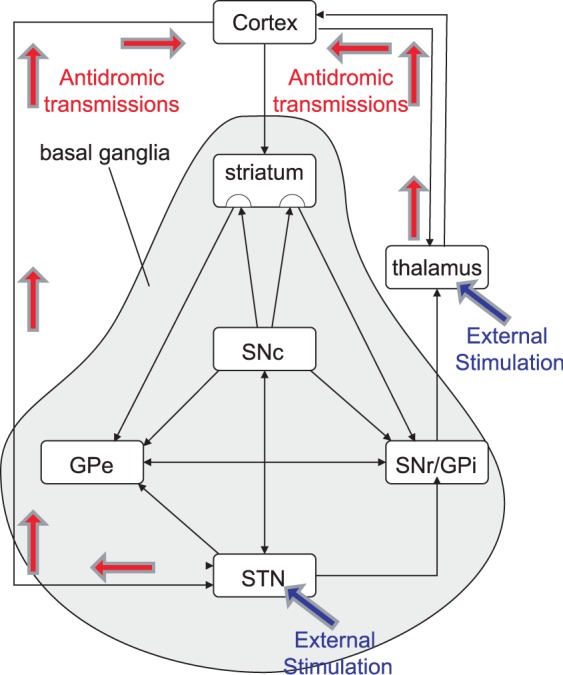
Cortical-basal ganglia-thalamo-cortical loop including the Tremor Ameliorating Targets (TAT): STN and thalamus.

Experiments have cast light on which axonal connections in the cortico-basal ganglia-thalamocortical circuit are essential to amelioration of tremor by DBS. The brain structures usually stimulated for this purpose are the ventral thalamus and the STN, which we will refer to as Tremor Ameliorating Targets (TATs). Their connections inside the cortico-basal ganglia-thalamocortical circuit are illustrated in Fig. 1. Using optogenetic methods, distinct circuits elements in freely-moving Parkinsonian model rodents were systematically driven or inhibited, showing that a similar therapeutic effect to stimulation of STN could be obtained by direct selective stimulation of afferent axons projecting from the cortex to STN [7]. That result, combined with the known importance of the cortex in commanding the cortical-basal ganglia-thalamo-cortical pathway [20], supports the notion that connections between the cortex and TATs are of critical importance in understanding tremor amelioration by DBS.

When we look at the form of activation, the results are more conclusive: spikes can travel in both directions from the stimulated axonal point, in the usual direction toward the synapses (orthodromically), and also toward the soma (antidromically). As spontaneous neuronal activity in basal ganglia has a lower frequency than the beneficial HF-DBS, it has been suggested that antidromic activation is the key mechanism in DBS [5]. Effective DBS stimulation frequencies are substantially higher than those thought to be used to encode information, and orthodromic excitation of downstream structures might therefore not be decoded by the neurons, but rather contribute by overriding pathological neuronal discharges [13]. Thus, there appears to be sufficient evidence to allow us to entertain the assumption that DBS achieves its beneficial effects by antidromic activation of long axons connecting different parts of the brain.

There are two main theories regarding the effect of the antidromic spikes: either (i) they facilitate the cortex, or (ii) they collide with ongoing cortical activation of the basal-ganglia or thalamus. The former hypothesis is based on suggestions that antidromic spikes activate cortical neurons [8], [9], [21], [22]. However, the correlation between the probability of antidromic somatic invasion and membrane potential shows that, at normal resting potential, the majority of spikes are filtered out of the cell body of cortical neurons [23]. We therefore turn our attention to the collisions of antidromic and orthodromic signals, assuming that this effect is more important than cortical facilitation – a view consistent with reviews of the literature [5], [13], [24].

**Figure 2:**
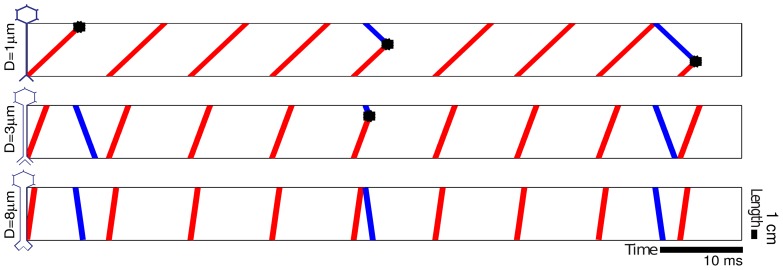
DBS antidromic blockade is less effective for axons with greater diameter. Interaction is shown between orthodromic beta spikes and an antidromic DBS pulse train in axons of different diameters. Beta somatic spikes at 29Hz are shown in blue traveling orthodromically (downward), while antidromic spikes due to high frequency DBS at 103Hz are shown in red. Velocities, distances, and pulse frequencies are in the physiologically and clinically appropriate ranges for the relevant pathways. The differing diameters result in differing conduction velocities (top to bottom: 9 m/s, 25.5 m/s, and 66.8 m/s) which results in a higher proportion of spikes clearing the axon without interference in larger-diameter axons.

We have outlined some assumptions required, and evidence in the literature, for the hypothesis that antidromic axonal activation due to DBS effectively blocks orthodromic transmission [5], [24], [25]. Our novel *slow axon antidromic blockade* (SAAB) hypothesis is a variant of this blockade theory: we hypothesize that axonal connections with large transmission times, i.e., slow axons, are preferentially blocked by antidromic activation of TATs. The mechanism is illustrated in Fig. 2. Note that whenever the axonal blockade is partial, the two hypotheses discussed above (facilitation versus collision) are not mutually exclusive. The plausibility of the facilitation hypothesis rests on the reliability of soma invasion. In fact, a partial blockade can reconcile the two hypotheses and provide a new way to look at the problem, in which cortical incoming signals are filtered or modulated by the probability of collision.

### The slow axon antidromic blockade hypothesis

Consider a cortical neuron projecting to the STN, with an axonal propagation delay between the soma and STN of 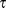. If the axon terminal in the STN is stimulated at time 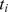, an antidromic spike would travel to the cortex and annihilate any spike it collides with in the axon in the interval 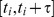. Since cortical activation also requires time 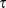 to reach the STN, this applies to any orthodromic spike initiated at cortex within the time range 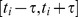. If consecutive DBS pulses in STN are delivered with an interval of 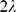, the probability of transmission (one minus the probability of blockade) of an orthodromic spike initiated at a random time can be computed by noting that there is complete blockade when 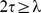:



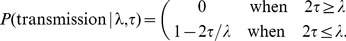
(1).

We now include the influence of the refractory period 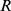, the time required by an excitable membrane to recover from an electrical pulse. During the absolute refractory period, a second stimulation pulse cannot evoke a spike in the membrane, while in the relative period a second spike is inhibited but not impossible. Interestingly, one of the proposed DBS working mechanism relies on this membrane property: the *depolarization blockade hypothesis* suggests that this refractory period is such that stimulated cells are not excitable between DBS spikes [26], [27]. Subsequent experiments showed that cells in the thalamus were able to fire at frequencies higher than [200] Hz when properly stimulated [28], implying refractory periods shorter than those assumed by that hypothesis.

In the SAAB hypothesis, this refractory period affects the transmission probability as follows:



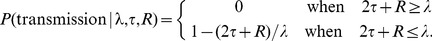
(2).

The new term is not as relevant as the effect of the delay of the travelling signal, since refractory periods in axons are smaller than in somas [19]. Notwithstanding this, estimates of axonal refractory periods, at which the second stimulating pulse was elevated by 50% with respect to the first pulse to elicit a spike, are in the range of 1.7–2.6 ms [28] or even as small as 0.5 ms [29]. We use the an intermediate value (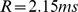) from the first of these studies [28].

Note that even in the case of refractory delays as large as 2.6 ms and with DBS stimulation of 130Hz, a complete blockade is only observed for delays greater than 2.5 ms. On the other hand, for refractory delays as small as 0.5 ms, only delays greater than 3.6 ms are completely blocked. Since latencies have been measured in the range 0.9–4 ms (see Methods section for details), even in these extreme scenarios, the blockade is only partial.

**Figure 3:**
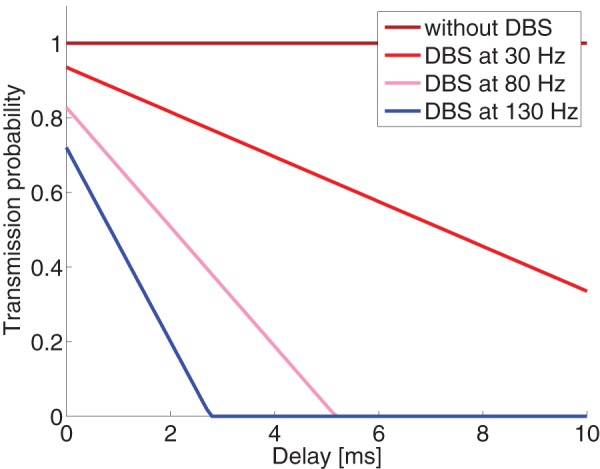
Transmission probability of a random orthodromic spike as a function of axonal delay, at different antidromic blocking frequencies. Computations here were based on equation (2). If we negglet the refractory period, the blockade is complete when the axonal delay exceeds one-half of the interval between antidromic spikes.

**Figure 4:**
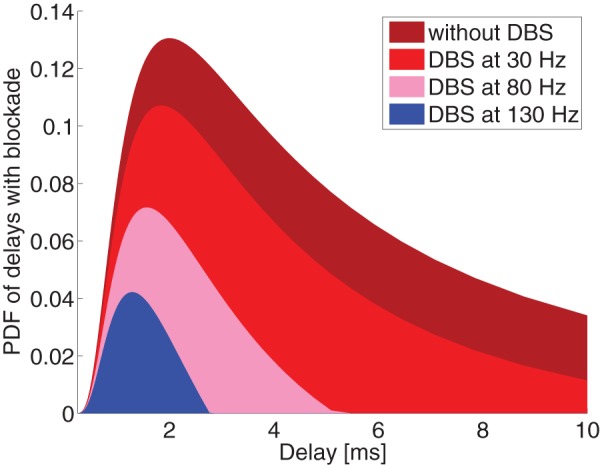
Distribution of axonal delays (in terms of Probability Density Function), as modulated by DBS. Higher frequency DBS dramatically shortens the distribution of delays.

Let us consider how the transmission probability of Eq. (2) changes under different axonal delays and DBS frequencies, as in Fig. 3. Here we observe that axons with the largest axonal delays (and hence smallest diameters) are blocked by high frequency DBS. It might appear that there is no substantial difference between stimulation at 80Hz and 130Hz, but this should be evaluated in light of the distribution of conduction latencies. The distribution of axonal diameters is fit empirically by a gamma distribution [30]. The relationship between axonal diameter and propagation velocity is well known; combining this with an estimate of axonal length leads yields a distribution of latencies (see Methods section for details). Fig. 4 shows the estimated distribution of latencies between between TAT and cortex, and show how this distribution would be affected by DBS at various frequencies. As can be seen, only frequencies larger than 130Hz block all transmissions with delays longer than 3 ms.

**Figure 5:**
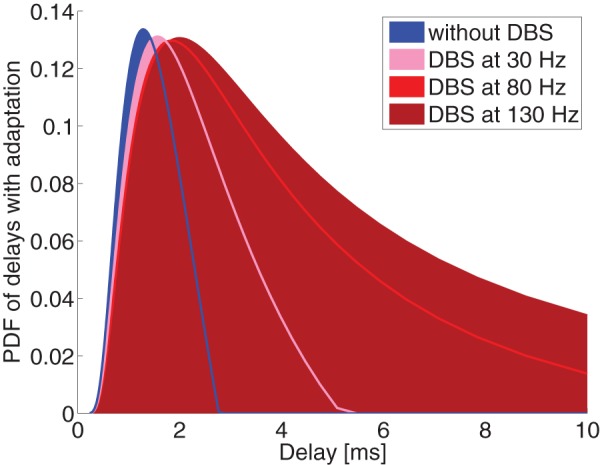
Distribution of axonal delays, as modulated by DBS, with gain adaptation operating to preserve the area under the curve.

It seems reasonable to assume that the brain will try to adapt the cortical activity to the external blockade by changing the excitatory postsynaptic potentials. We assume that synaptic efficacies are up-regulated to maintain roughly the same total postsynaptic activity. Such scaling effect could however be achieved in a variety of ways (see [31] and references therein), but there is evidence for these sorts of adaptive gains throughout the nervous system, including in particular in the motor control loop [32]. The result of this, depicted in Fig. 5, is a reduction of the mean delay in the motor control loop without a substantial decrease in its total gain, which in turn serves to stabilize the feedback system, thus ameliorating tremor.

### Effect in the motor loops

**Figure 6:**
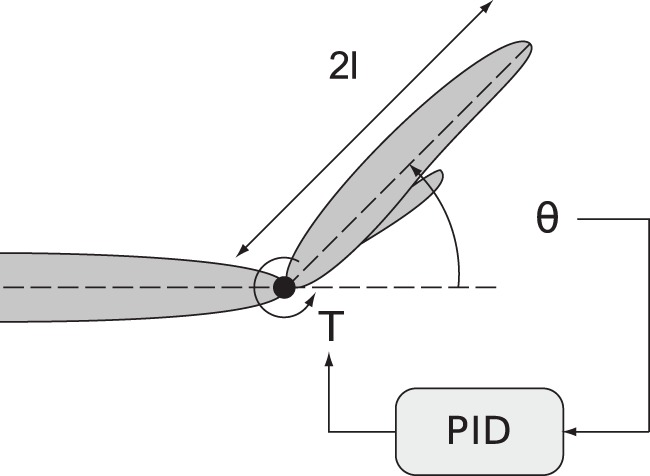
A simple biomechanical model of a hand.

We use a basic control model to argue that reducing the effective delay of the feedback loop has two effects observed in experiments: decrease of the tremor amplitude and increase of its frequency. It is well known in control theory that a communication delay in the feedback path of a control system can have a destabilizing effect [33]. This in illustrated in Fig. 6, which shows a simple biomechanical model of wrist angle under the action of torque 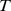 produced by a feedback control circuit. We assume that the control circuit uses a generic control structure (PID, or proportional, integral plus derivative [34]) to maintain the hand in a horizontal position against gravity (See method's section for details).

**Figure 7:**
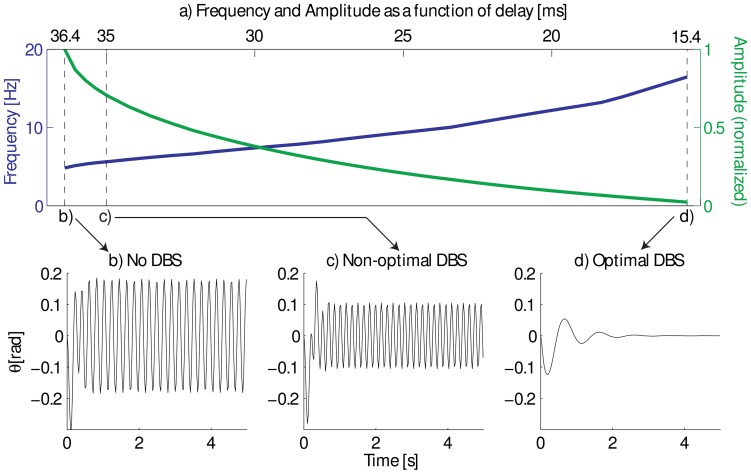
Closed-loop control is used to regulate wrist angle at the horizontal position 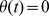 with control gains selected to reproduce the mean measured amplitude and frequency [2]. Panel (a) shows how the frequency of the oscillation increases and the amplitude decreases when reducing the delay. Panels (b)–(c) show different PD tremor at different conditions: (b) no DBS, (c) a non-optimal DBS, and (d) optimal DBS. (Normal physiological tremor usually ranges between 6–15Hz [36].)

Fig. 7 shows the behavior of the closed-loop system as a function of the delay parameter. Simulations show how the controller attempts to maintain a the horizontal position when the support is removed and gravity starts acting, as is popular in experimental studies [2], [35]. First we calculate the controller gains to reproduce the measured mean amplitude and frequency [2] in Parkinsonian patients under a feedback delay [36]. The dynamics of this experiment are depicted in Fig. 7b. When the delay is reduced to 35 ms, the amplitude and frequency predicted by the model match those results measured in PD patients milliseconds after the device is turned on (Fig. 7c). In a third simulation, we further decrease the delays and the model predicts a behavior typical in normal physiologic tremor or in tremor under DBS after several seconds of stimulation [36], as shown in Fig. 7d.

As can be seen in Fig. 7a, the model shows that both amplitude and frequency depend upon the value of the delay parameter in a predictable manner, with a larger delay leading to a lower frequency and a higher amplitude. This behavior is characteristic of a well known phenomenon in the theory of dynamical system known as a (supercritical) Hopf bifurcation [37], the same bifurcation observed in the models simulating the competition between feedback loops in the BG [38]. We note that the stable regime is finite: delays beyond a certain critical value lead to a bifurcation that renders the oscillations unstable. This phenomenon is also extremely robust to the particular details of the controller. In fact, normal physiologic tremor can also be obtained for different delay values by selecting proper controller gains, but still with the same change of behaviour as shown in Fig. 7a.

We should note this hypothesis does not assume that the typical dopamine deficit in PD increases latency in the motor control loop, although it is certainly consistent with that notion. It is however logically possible that decreasing motor control loop latency could serve to stabilize an unstable motor control loop which has been rendered unstable in some other way. This notion would agree with computational models [39] and with the observation that the drug levodopa, commonly used to treat PD, suppresses tremor but keeps the frequency invariant, probably by changing the gains between the direct and indirect pathways in the BG [2], [35], [40]

### Discussion: testable predictions for SAAB

In addition to explaining previous experiments, a new hypothesis should be testable and falsifiable. As discussed above, the SAAB hypothesis is unique in that it naturally accounts for a variety of observed phenomena, including the pulse frequency range effective in DBS and the clinical effect of slightly sub-therapeutic DBS stimulation frequencies. We now explore a variety of testable novel predictions made by this hypothesis.

It is possible to measure the ADDs (Axonal Diameter Distributions) [30] and pathway lengths to test the following predictions. (a) Bundles of axons traveling from the cortex to the TATs should have similar delay distributions, i.e., similar relationship between the length and the diameter and even with the degree of myelination among different mammalian brain sizes.(b) Where there are substantial differences in the minimum effective DBS frequency, there should also be differences in the delay distribution of the stimulated pathway. (If this observation were confirmed, pre-clinical studies could estimate the optimal stimulation frequency, or even other DBS locations, prior to DBS electrode implantation.) (c) If a patient has a narrower axonal delay distribution, DBS is less likely to be effective.

We hypothesize that DBS reshapes the impulse response of the relevant cortical-basal pathway. This distribution of delays, and its modulation by DBS, could be directly measured by transcranial magnetic stimulation in concert with stimulation of an implanted electrode. Such modulation might also be measured by short-term cross-correlations between time-domain recordings of activity in cortex and TATs. In fact, cortex response to DBS has been measured with electroencephalograms, and it was found that the amplitude of cortical events due to antidromic activation decreases as the frequency of stimulation increases [8], [9]. Assuming that somatic invasion of antidromic spikes is a reliable mechanism, this result supports the SAAB hypothesis, since higher DBS frequencies would block more fibers. Also, the motor control loop impulse response can be directly measured by mechanical perturbation of a load during a motor control task, which would allow modulation of the motor control impulse response by DBS to be observed.

Interesting predictions are also obtained by the ability of the SAAB hypothesis to explain experiments where DBS of the spinal chord suppresses tremor [41]. These results have two noteworthy features: (a) the frequency of stimulation is more than double that in usual TATs (300Hz); and (b) the electrode is located in the sensory fibers of the spinal cord and not in the normal DBS targets. First, some of the spinal cord sensory fibers go to the cortex via the brainstem. These axons share common segments with the axons connecting the thalamus and the cortex [23]. Second, since the stimulation frequency is between two and three times higher than that usual in conventional TATs, the shared pathway should have associated delays between two and three times shorter than the thalamus-cortex pathway. Both predictions are testable.

We have presented crisp predictions, which would serve as fingerprints of a slow axon antidromic blockade. It is important to note that the SAAB hypothesis does not imply that no other mechanism can ameliorate tremor, nor does it imply that SAAB is the only mechanism by which DBS ameliorates tremor. In fact, in future work we would like to extend the hypothesis to include the effect of cortical facilitation and orthodromic spikes. In a more speculative vein (a) other pathological oscillatory motor behaviour, such as stuttering, might also be ameliorated by a selective blockade of slow axons in the relevant pathways, and (b) other conditions for which treatment by DBS has enjoyed success, such as depression [42], might involve SAAB.

## Methods

Our hypothesis is based in the well-known fact in neurology: that long myelinated axons conduct traveling spikes at different velocities, and that those velocities are proportional to the axonal diameter. A literature search found no reports which directly measured such distributions in the pathways between the cortex and TAT. On the other hand, a very simple model with the essential elements of the hypothesis was used to check and illustrate the SAAB hypothesis. In this section we first describe the method used to estimate the conduction velocity and then give an outline of the simple biomechanical model.

### Estimation of axonal propagation delays in motor pathways

In order to test the plausibility of the SAAB hypothesis, we need to estimate the distribution of axonal delays between the TAT and the cortex. A recent work gathering information about axonal conduction delays [43] includes a table with axonal delays and velocities for several mammalian species. Although there are important differences among different axonal pathways, minimal conduction times of homologous pathways are quite similar among brains of dramatically different sizes. This agrees with the hypothesis that the distribution of axonal diameters in white matter are scaled to preserve similar minimum delays in homologous pathways, independently of brain size [44]. Unfortunately, this review does not provide information about the motor Cortico-thalamic and Thalamo-cortical loops.

Based on the assumption that minimum delays are not affected by brain size, we have found several experimental works in the literature where similar delays have been measured between the cortex and the rat STN [21], human STN [29], and mouse thalamus [25]. The most common measurement of latency in the connections relevant for the hypothesis is 2 ms [21], [25], [29], although in some works latencies as slow as 4 ms [25] or as fast as 0.5 ms [21] have also been observed. Examination of the literature revealed only one group measuring latencies consistently less than 2 ms in human STN [8] and human thalamus [9] of 0.6 ms–1.4 ms and 0.7 ms–1.1 ms respectively. However, these experiments were conducted: (i) without anesthetics; and (ii) in conscious patients where the brain was not exposed to recording instruments. Each of these conditions may change neuronal conduction velocity. In addition, following the SAAB hypothesis, we predict the DBS blockades slow axons and therefore that these measurements primarily observe the fast axons.

To summarize: despite a variety of reports of studies measuring axonal delays, there was insufficient information in these studies to directly estimate the *distribution* of delays. Probability density functions of axonal diameters, however, have been studied and are usually approximated by a gamma distribution
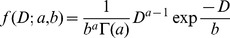
(3)where 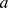 and 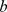 are the so-called shape and scale parameters, that should be estimated from available measurements, and 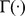 is the gamma function [30]

It is well known that in mylenated axons conduction velocities are linearly related to to axonal diameter. Here we use the numeric values from one particular report of the empirical relationship between propagation velocity and axonal diameter [45]:
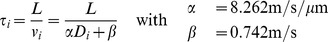
(4)where 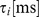, 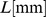, 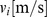, and 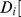 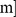 are the travel times, length, velocities, and axonal diameters, respectively, and 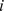 indexes particular axons. The parameters 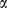 and 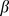 describe the linear relationship found between velocity and diameter, including the correction factor for the shrinkage of the axonal diameter after fixing and embedding the tissue in paraffin.

The distribution of latencies can thus be derived from equations (4) and (3).
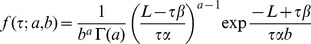



**Figure 8:**
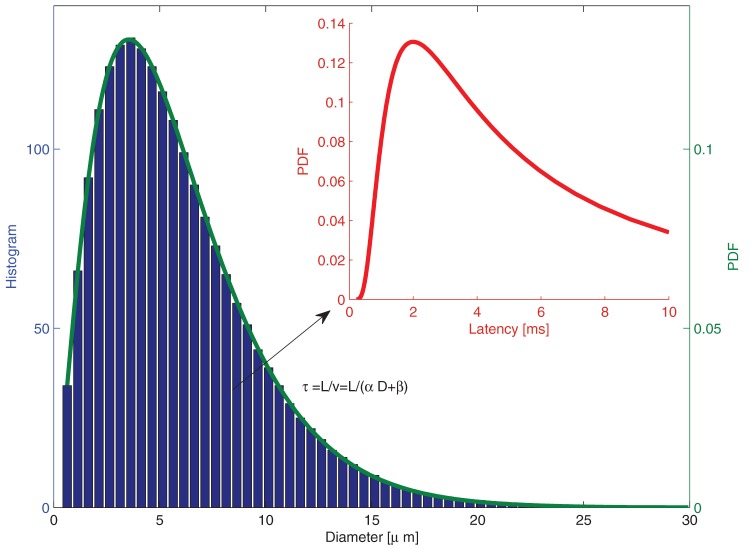
Assumed delay distribution. The delay distribution is calculated by assuming a mode of 2[30].

Taking into account the latencies reported in the literature, we take the most common latency to be 2 ms (the mode of the distribution) and the variance to be such that the longest and smallest latencies measured have probability greater than 0.1 (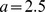, 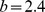). For clarity let us focus on the path between the STN and cortex. Its axonal length is approximately 6 cm [8] and the resulting distribution of delays can be seen in Fig. 8, where we have represented only the part of the distribution with delays less than 10 ms. Note that similar results should be obtained for the thalamus, the only difference being that as the length is approximately one centimeter shorter [9], axons should be thinner on average to result in similar axonal delays.

### Biomechanical model

A simple biomechanical model of the motor control loop is employed to illustrate and check the main characteristics of the hypothesis (Fig. 6). The equations of motion of this model are
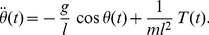
(5)where 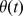 denotes the wrist angle as a function of time, 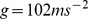 is the local acceleration due to gravity, 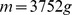 is the mass of the hand, 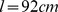 is the distance from the joint to the center of mass, and 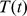 is the applied torque. (Actual measured hand mass and arm lengths are typically 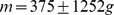 and 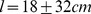, respectively). We assume that the torque exerted is a control force, of the form
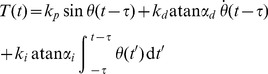
(6)where 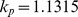, 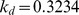, 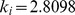 are the proportional, derivative and integral controller gains and 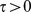 is a fixed delay associated with motor circuit control processing. The function 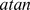 models saturation and 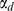 and 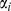 are scaling factors.
