# Correction: A Slow Axon Antidromic Blockade Hypothesis for Tremor Reduction via Deep Brain Stimulation

**DOI:** 10.1371/journal.pone.0106145

**Published:** 2014-08-13

**Authors:** The PLOS ONE Staff

There is an error in Equation 1. Please see the corrected Equation 1 here.



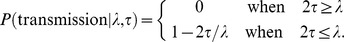



The first paragraph of the “Biomechanical Model” sub-section of the Methods contains multiple errors. The correct paragraph is: A simple biomechanical model of the motor control loop is employed to illustrate and check the main characteristics of the hypothesis (Fig. 6). The equations of motion of this model are



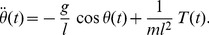
(5)where 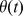 denotes the wrist angle as a function of time, 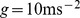 is the local acceleration due to gravity, 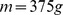 is the mass of the hand, 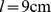 is the distance from the joint to the center of mass, and 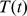 is the applied torque. (Actual measured hand mass and arm lengths are typically 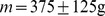 and 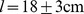, respectively).

The image for Figure 5 is incorrect. Please see the complete, corrected Figure 5 here.

**Figure 5:**
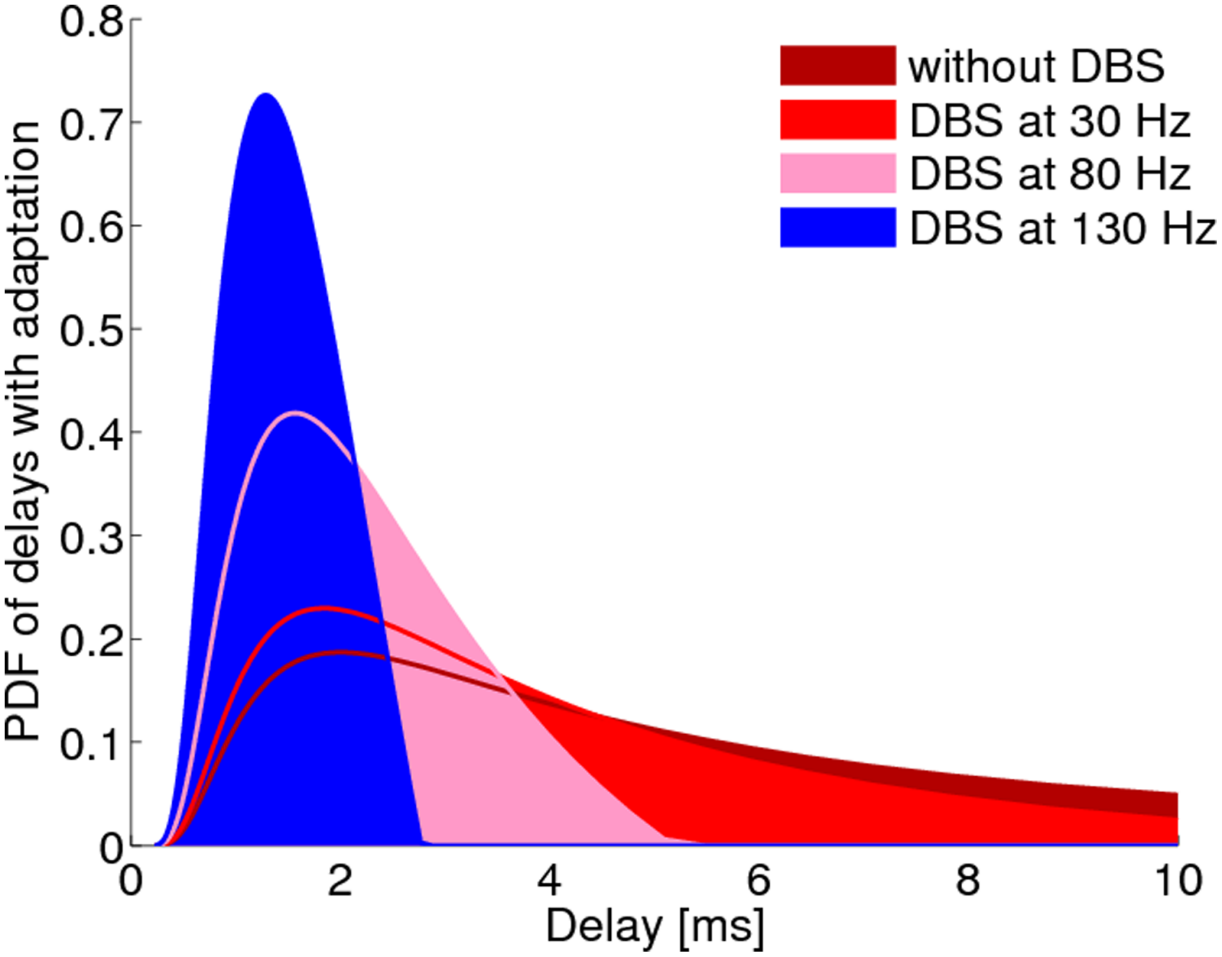
Distribution of axonal delays, as modulated by DBS, with gain adaptation operating to preserve the area under the curve.
